# Consumer evaluation of complaint handling in the Dutch health insurance market

**DOI:** 10.1186/1472-6963-11-310

**Published:** 2011-11-15

**Authors:** Sonja Wendel, Judith D de Jong, Emile C Curfs

**Affiliations:** 1 NIVEL, Netherlands Institute for Health Services Research, P.O. Box 1568, 3500 BN Utrecht, the Netherlands; 2 Open University, School of management, P.O. Box 2960, 6401 DL Heerlen, the Netherlands

## Abstract

**Background:**

How companies deal with complaints is a particularly challenging aspect in managing the quality of their service. In this study we test the direct and relative effects of service quality dimensions on consumer complaint satisfaction evaluations and trust in a company in the Dutch health insurance market.

**Methods:**

A cross-sectional survey design was used. Survey data of 150 members of a Dutch insurance panel who lodged a complaint at their healthcare insurer within the past 12 months were surveyed. The data were collected using a questionnaire containing validated multi-item measures. These measures assess the service quality dimensions consisting of functional quality and technical quality and consumer complaint satisfaction evaluations consisting of complaint satisfaction and overall satisfaction with the company after complaint handling. Respondents' trust in a company after complaint handling was also measured. Using factor analysis, reliability and validity of the measures were assessed. Regression analysis was used to examine the relationships between these variables.

**Results:**

Overall, results confirm the hypothesized direct and relative effects between the service quality dimensions and consumer complaint satisfaction evaluations and trust in the company. No support was found for the effect of technical quality on overall satisfaction with the company. This outcome might be driven by the context of our study; namely, consumers get in touch with a company to resolve a specific problem and therefore might focus more on complaint satisfaction and less on overall satisfaction with the company.

**Conclusions:**

Overall, the model we present is valid in the context of the Dutch health insurance market. Management is able to increase consumers' complaint satisfaction, overall satisfaction with the company, and trust in the company by improving elements of functional and technical quality. Furthermore, we show that functional and technical quality do not influence consumer satisfaction evaluations and trust in the company to the same extent. Therefore, it is important for managers to be aware of the type of consumer satisfaction they are measuring when evaluating the handling of complaints within their company.

## Background

The new Dutch healthcare system, launched on January 1st 2006, is being constantly scrutinized by policy makers and researchers from various industrialized countries [1-5]. Central to the new healthcare system is the concept of managed competition, which combines competition between health insurers and providers of health care with regulation such as the obligation for insurers to accept all applicants for the basic insurance package. The new system consists of universal health care coverage, the individual mandate, combined with an optional private supplementary insurance.

In this new system, the health insurer and the individual Dutch citizen have taken on new roles. Health insurers are responsible for negotiating with care suppliers about the quality of care provided to their clients as well as about the price of care. Furthermore, health insurers have the freedom to selectively contract care suppliers if a care provider does not fulfil the quality standards or if prices are too high [6,7]. Individuals in the Netherlands are required to purchase a basic insurance package from a healthcare insurer of their choice and have the option of purchasing supplementary insurance for additional healthcare not included in the basic insurance package, either from that same insurer or a different one.

In addition to being free to choose whichever healthcare insurer one would like, consumers are also able to change their insurance plan or even switch their insurer every year if they are dissatisfied with the service offered or can get a better offer elsewhere. Reitsma-van Rooijen, Brabers and de Jong [8] show that consumers' satisfaction with the service of their current insurer is an important factor to stay insured with that insurer. In the Netherlands, consumers are able to rate the service quality of their health insurer on various aspects and this information is publicly available on the Internet (e.g., http://www.kiesbeter.nl or http://www.independer.nl). A study investigating a number of service quality aspects (e.g., if clients are treated with courtesy or if they received the right information) of 13 Dutch insurance companies consisting of 31 labels illustrates that the health insurer fulfils most of the service aspects at all times [9]. This study also includes an overall rating of health insurers by their enrolees, which illustrates that health insurers are rated high (between 7.5 - 8.5 on a scale from 1 to 10 where 10 is the highest score).

Thus, the focus on consumers is one key element of this new system. This in turn has consequences for the healthcare insurance market since insurers are now confronted with intensified competition [3-5]. Insurers compete on the premium of the insurance package and the service they are offering [10]. Consequently, service quality and consumer satisfaction are becoming increasingly important issues for healthcare insurers. They need to develop and maintain a good relationship with their customers and to differentiate themselves from competitors.

Also, the trust that customers have in their healthcare insurer is a crucial element and contributes to maintaining lasting relationships [11]. There has been increased attention of trust in the context of healthcare and specifically trust of enrolees towards their health insurer in the Netherlands [12,13]. Satisfaction and trust are thus two key marketing related factors that companies can focus on to build successful long-term relationships with its customers.

The effective management of complaints is a particularly challenging aspect in managing the quality of the service. Smith and Bolton [14,15] stated that handling complaints effectively can even increase consumer satisfaction beyond the level before the failure occurred. Despite the fact that companies seem to be aware of the significance of effective handling of complaints, there is plenty of evidence across various industries that companies' management of complaints is poor [16-18]. As Estelami [17] points out, about half of the complaining customers are not content with the way their complaints are handled. Also Friele and Sluis [18], who investigated the situation in hospitals, point out that in health care many patients are dissatisfied with the way their complaints are handled. Regarding trust, most studies have specifically focused on the relationship between trust towards their health insurer and selective contracting and channelling of enrolees towards healthcare providers [13]. Yet, little consideration has been given to the relationship between trust and service quality of health insurers in a complaint handling situation, which is also relevant for other countries that have adapted a system of managed competition.

Therefore, the focus of our study is twofold. First, we test a model showing the impact of the quality of service on consumer complaint satisfaction evaluations and trust in a company in the context of the Dutch health insurance market. Second, we extend this model by hypothesizing about the relative effects of the quality of service on consumer complaint satisfaction evaluations and trust in a company.

### Service quality

Medical practitioners, like private sector managers, as well as academics highlight the importance of effectively handling consumer complaints, as part of the service being offered. This strongly affects customer or patient satisfaction [19,20] and in turn the profitability of a company [21]. Furthermore, there is evidence in literature that the quality of service is an important determinant of customer satisfaction [14,22-24]. The concept of service quality has been explored extensively in the literature and various models have emerged [25]. One of the most prominent models is the functional and technical quality framework by Grönroos [26]. According to this model, service quality consists of two dimensions, functional quality and technical quality. Functional quality refers to *how *the service is being delivered. In particular, it refers to how customers perceive the interaction as well as the fairness of the process during service delivery [24,27]. Functional quality covers, for instance, the responsiveness of the company to a customer's complaint and the friendliness of the service personnel. Technical quality addresses the *what *question and reflects customers' perceptions of the outcome they receive such as refunding money or the cleanliness of a hotel room [27].

Justice theory, which predicts consumers' reactions to conflict situations (e.g., filing of a complaint) provides additional support for this model. Justice theory puts forward that people want to be treated fairly with respect to the outcome they receive as well as the interaction and the process they experience. Since one can interpret these different types of justice as quality dimensions of complaint management [28] we also rely on justice theory literature in this paper.

### Complaint satisfaction evaluations

**Figure 1:**
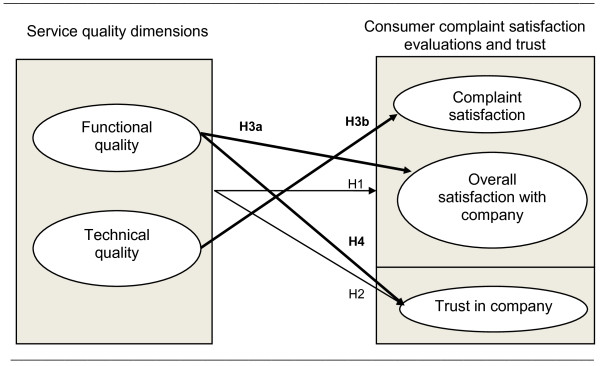
**Conceptual model**. Besides the hypothesized direct effect of service quality dimensions on consumer satisfaction evaluations and trust (H1 and H2), we hypothesize that there are relative effects of service quality dimensions on consumer satisfaction evaluations and trust (H3 and H4). These effects are highlighted in **bold **and indicate the service quality dimension that better predicts complaint satisfaction, satisfaction overall with company or trust in the company.

In our conceptual model (see Figure 1) we make a distinction between two different satisfaction outcome variables. These are consumer complaint satisfaction after the complaint; and consumer satisfaction overall with the company after the complaint [23,28]. The former, consumer complaint satisfaction after the complaint, hereafter named complaint satisfaction, is defined as 'the degree to which the complainant perceives the company's complaint-handling performance as meeting or exceeding his or her expectations' [19]. Consumer satisfaction overall with the company after the complaint, hereafter named overall satisfaction, is defined as 'the degree to which the complainant perceives the company's general performance in a business relationship as meeting or exceeding his or her expectations' [19]. It should be noted that the latter is cumulative and therefore relates to more than just one transaction that took place between a customer and the company. Consumer complaint satisfaction in contrast is a measure of satisfaction specific to one incident such as a service failure.

### Trust in the company

We investigate trust in the company (after complaint handling) as a third important outcome variable. Whereas satisfaction with the company is based on past experiences, trust is a concept measuring more expectations about how the other party will perform in the future [29,30]. Existing research illustrates the importance of trust as an influential driver for building successful long-term relationship between a company and its customers and also for driving profitability of a company [11,31]. Studies also show that satisfaction with the service being offered directly effects customers' trust in the company [11,32]. We define trust as 'customers' confidence in an exchange partner's reliability and integrity' [31] and 'can be relied on to deliver on its promises' [33].

### Theory and hypotheses

#### Main effects of quality dimensions on consumer satisfaction evaluations

We apply the functional and technical framework and put forward the view that functional and technical quality affect evaluations of consumer satisfaction. Several studies provide evidence that functional and technical quality directly influence consumer complaint satisfaction as well as overall satisfaction with the company [23,34]. Maxham and Netemeyer [23] for instance, explored the effects of consumers' perceived justice with the recovery following a banking service failure, on consumer complaint satisfaction and found support for these effects. Lassar and his coauthors [14] compared the most prominent concepts of service quality in the private banking sector and found evidence that functional and technical quality are reliable in predicting overall satisfaction. Therefore, we hypothesize:

H1: Functional quality and technical quality positively influence (a) consumer complaint satisfaction and (b) consumer overall satisfaction with the company.

#### Main effects of quality dimensions on trust in the company

In addition to measures of consumer satisfaction, we also expect to find a relationship between functional and technical quality in the trust felt in a company. As mentioned, the importance of trust in a company is highlighted as a key contributor to maintaining valuable long-term relationships with customers [11,31]. The importance of service quality dimensions as drivers for building these trusting long-term relationships as well as a means for differentiation from competitors has been stressed in literature [11]. For example, the effects of functional and technical quality on the trust in the adviser of financial planning services have been explored in previous research [32]. They found that the greater the effect of functional and technical quality, the stronger the trust. Thus, we argue that this relationship also holds true in the context of our study; if consumers perceive a high functional and technical quality regarding complaint handling, then they will also have more trust in the company. This leads to the following hypothesis:

H2: Functional quality and technical quality positively influence trust in the company.

### Relative effects of quality dimensions on consumer satisfaction evaluations

We also expect that there will be a difference in the size of the effect between the two service quality dimensions and measures of consumers' satisfaction. It has been documented in literature that functional elements of the service are mostly related not only to a specific transaction that took place during service delivery, such as handling a complaint, but also capture the perception consumers have of a company over a longer period of time [23,35]. Yet, elements of the service related to the outcome, that is the technical quality, are more related to a specific transaction that took place, for example the last service failure [23]. Based on this theoretical approach, we expect that functional quality is a better predictor for capturing overall consumer satisfaction, whereas technical quality is better for measuring satisfaction when referring to a specific transaction (i.e., consumer complaint satisfaction). Therefore, we hypothesize that:

H3a: There will be a stronger effect of functional quality as compared to technical quality on consumer overall satisfaction.

H3b: There will be a stronger effect of technical quality as compared to functional quality on consumer complaint satisfaction.

### Relative effects of quality dimensions on trust

Moreover, we expect in the specific service context we are investigating, that functional quality will be a better predictor of trust in the company than technical quality. It has been documented in literature that the service context, for example a service with low versus high contact, might have an influence on the effect of functional and technical quality on trust in a company [11]. A reason might be that the outcome for some services might be difficult to evaluate by consumers and therefore, consumers focus more on the functional elements of service delivery. This rationale might also be valid for the setting of our study. Specifically, consumers might have difficulties evaluating the fairness of the outcome of their complaint, and as a result might pay more attention towards the functional elements, as they appear more pertinent when evaluating the company.

H4: There will be a stronger effect of functional quality as compared to technical quality on consumer trust in the company.

## Methods

### Design of the study

Our empirical study was undertaken among members of a large health insurance panel in the Netherlands. The panel was set up in 2006 by the Netherlands Institute for Health Services Research in co-operation with one of the biggest healthcare insurers in the Netherlands. At the time this study was undertaken the panel consisted of about 7600 members. On average, panel members are invited three times a year to participate in research, which is related to various topics in the field of health care. Here, the study population consisted of members who had complained to their healthcare insurer within the last 12 months at the time this study was undertaken. Since these complaints were registered at the insurance company, we matched respondents that had filed a complaint with their insurer with members of the health insurance panel.

This way, we identified 184 members who had complained to their healthcare insurer and received 150 usable questionnaires (response rate of 81.5%), 58% of the responders were men. The average age is 66.2 years ranging from 29 to 92 years. The majority of respondents (90%) are more than 10 years insured at their insurer and the majority of the complaints (53%) were cost-related in that clients were not completely or not at all reimbursed for costs incurred. See Table 1 for an overview of the sample characteristics. Additionally, there are no differences between respondents and non-respondents regarding age and only a slight difference regarding gender (50% men in non-respondent group).

**Table 1:** Sample composition (n = 150)

	*Mean (SD)*	
**Age**	66 (11.7)	
	*n *	*%*
**Sex**		
Female	63	42%
Male	87	58%
		
Number of years insured at insurer*		
Between 2-4 years	3	2.2%
Between 5-10 years	11	8.2%
> 10 years	120	89.6%

* 16 respondents did not provide an answer to this question

The data were collected by means of a postal questionnaire. The protection of the collected data from the insurance panel was laid down in privacy regulations, safeguarding ethical consent, and registered by the Dutch Data Protection Authority (nr. 1309664). The questionnaire contained multi-item measures adopted from existing studies to measure the core variables. Furthermore, the questionnaire was administered in Dutch. Here, we made use of a double-back translation procedure by a qualified translator. Some of the items had to be slightly adapted to suit the context of our research.

### Measures

Each of the items was measured by means of a 7-point Likert-type scale (strongly disagree = 1 to strongly agree = 7). The following three *dependent variables *were identified: (1) complaint satisfaction, (2) overall satisfaction with the company, and (3) consumer trust in the company. Consumer complaint satisfaction and overall satisfaction with the company are both composed of 3 items as used by Homburg and Fuerst [19]. Trust in the company is assessed by 4 items based on a scale by Eisingerich and Bell [11]. Table 2 provides an overview of the scale items used for each construct and descriptive information. The two *independent variables *functional quality and technical quality were operationalized based on a number of selected sources that suited the context of our study. Specifically, functional quality was assessed with 8 items based on scales by Homburg and Fuerst [19], Maxham and Netemeyer [23], Vorhees and Brady [20] and Tax, Brown and Chandrashekaran [36]. Technical quality was operationalized based on 2 items by Vorhees and Brady [20] and 1 item by Maxham and Netemeyer [23].

**Table 2:** Scale items and descriptives for construct measures (N = 126 for factor 1-3, N = 101 for factors 4 and 5)*

*Construct *	*Items*	*n*	*Mean (SD)*	*Loadings*	*Average variance extracted*	*Composite reliability*	*Alpha *
Factor 1 Complaint satisfaction	I was satisfied with the complaint handling of company X**	141	4.2 (2.2)	0.76	0.70	0.87	0.87
	I had a positive experience when complaining to company X	138	4.1 (2.0)	0.77			
	I am not satisfied with the handling of my complaint (R)^†^	136	3.8 (2.2)	0.73			
Factor 2 Overall satisfaction with company	Overall, to purchase a healthcare insurance from company X was a good decision	136	5.3 (1.4)	0.77	0.67	0.86	0.91
	Overall, I am satisfied with company X	140	5.4 (1.4)	0.82			
	Overall, so far, I have had positive experiences with company X	142	5.4 (1.4)	0.60			
Factor 3 Trust in the company	Company X is an organization that can be trusted at all times	139	5.2 (1.4)	0.76	0.67	0.89	0.94
	Company X is an organization that is honest and truthful	137	5.1 (1.4)	0.80			
	Company X is an organization that can be counted on to do what is right	136	4.9 (1.6)	0.71			
	I have confidence in company X as an organization	139	5.3 (1.4)	0.66			
Factor 4 Functional quality	The employee understood exactly my problem	122	5.1 (1.8)	0.60	0.55	0.91	0.89
	The employee treated me in a courteous manner	120	5.8 (1.1)	0.67			
	The employee was honest in dealing with me during the encounter	117	5.2 (1.6)	0.61			
	The employee seemed to be interested in my problem	119	4.9 (1.7)	0.79			
	The employee seemed concerned about my problem	115	4.5 (1.9)	0.76			
	Company X responded quickly to my complaint	138	3.7 (2.2)	0.65			
	Company X gave me the opportunity to explain my point of view of the problem	138	4.9 (1.9)	0.57			
	Overall, the company's complaint handling procedure was fair	135	4.8 (1.6)	0.55			
Factor 5 Technical quality	The outcome I received was fair	136	4.3 (2.3)	0.90	0.90	0.97	0.94
	Given the inconvenience caused by the complaint, the outcome I received from company X was fair	131	4.1 (2.3)	0.91			
	The outcome I received was right	134	4.2 (2.3)	0.93			

* We only used data of the cases that provided answers to all items

**7-point Likert-type scale used (Strongly Disagree [1] to Strongly Agree [7])

† R = reversed item

## Results

### Construct validation

In order to validate our constructs we made use of factor analysis. Since the dependent and independent variables address distinct concepts, we first performed a factor analysis related to consumer satisfaction measures and trust in the company. The second analysis included items related to technical and functional quality. For these analyses we only used data of the cases that provided answers to all items. We used the rotated (varimax) results and evaluated the eigenvalues, scree plots, and explained variance to extract appropriate and reliable items. Based on this evaluation we identified three factors for the first factor analysis - complaint satisfaction, overall satisfaction, and trust in the company - which accounted for more than 95% of the total variance. Two factors were identified for the second factor analysis - functional and technical quality- these accounted for more than 95% of total variance (Table 2). To validate our measures further, we evaluated content validity, construct validity (i.e., convergent and discriminant validity) and composite scale reliability. In order to assure content validity, two experts working in the field of complaint management had evaluated the survey before it was sent out. One expert who is familiar with the processes of complaint management at the insurer evaluated the survey and a second expert who is familiar with complaint management literature assessed the survey. This did not result in the need for any content-related adaptations; only wording adaptations were made. To assess convergent validity, we investigated the loadings of each item on their respective scale and found that all items converge on the appropriate scale (Table 2). In order to evaluate whether an item correlates more strongly with its own factor than with any other factor (discriminant validity), we followed an approach by Fornell and Larcker [37], which entails that the square root of the average variance extracted (AVE) should be higher than the inter-correlations with the other scales identified in the model. Therefore, we first computed the AVE of each scale as follows: AVE = (sum of squared standardized loading)/(sum of squared standardized loading + sum of indicator measurement error). We found that the square root of the AVE of each scale exceeds all inter-correlations with other scales and also exceeds the cut-off value of 0.50 [37]. Table 3 displays the correlation matrix of the constructs. Next, we examined the composite reliability (CR; overall scale reliability measure) by applying the following formula: CR = (sum of standardized loading) 2/(sum of standardized loading) 2 + sum of indicator measurement error). The results show that the CR of all scales exceeds the cut-off value of 0.70. Finally, we examined the Cronbach's alpha values, which ranged from 0.87 to 0.94 (Table 2). Based on these results, we can conclude that our scales are adequate for further analysis and consequently we took the average of the appropriate scale items for each factor. In taking the average of the scale items we also included the cases that did not provide an answer to all items and we included the following restriction: respondents must have answered at least two questions for each of the following factors: complaint satisfaction, overall satisfaction with the firm, trust in the company, and technical quality. With respect to functional quality at least three questions had to be answered. We chose these restrictions based on the number of items that each construct contained. We further performed three regression analyses on: the complaint satisfaction on technical quality and functional quality; the overall satisfaction on technical and functional quality; and lastly, the trust with the company on technical and functional quality.

**Table 3:** Correlation matrix of constructs

	Complaint satisfaction	Overall satisfaction with company	Trust in the company	Functional quality	Technical quality
Complaint satisfaction	1.0				
Overall satisfaction with company	0.47	1.0			
Trust in the company	0.57	0.8	1.0		
Functional quality	0.65	0.56	0.61	1.0	
Technical quality	0.84	0.35	0.47	0.54	1.0

### Model results

Table 4 shows the results of our regression analyses. Here, we report the coefficient (b), the standardized betas (B) and the p-value. Our models explain a moderate (32% and 43% for overall satisfaction and trust in the company respectively) to a high amount of total variance (76% for complaint satisfaction). The results further illustrate that there is a positive effect of functional and technical quality on complaint satisfaction (*p *< 0.001). We find too that functional quality significantly affects overall satisfaction (*p *< 0.001). Lastly, we identified significant effects of functional quality on trust in the company (*p *< 0.001) and of technical quality on trust in the company (*p *= 0.011). Yet, we do not find a significant effect of technical quality on overall satisfaction. Thus, all, but one of the direct relationships put forward are supported. Examining the standardized betas to observe the magnitude of the effect, the results show that there is a stronger effect of functional quality on overall satisfaction (beta = 0.53) than on complaint satisfaction (beta = 0.28). We also examined the relative effects of functional and technical quality on trust in the company. As hypothesized, there is a stronger effect of functional quality on trust in the company (beta = 0.52) as compared to technical quality on trust in the company (beta = 0.21). We cannot compare the relative effect sizes of technical quality on satisfaction evaluations since there is no significant direct effect of technical quality on overall satisfaction. Examining the coefficients (b), we can also state that we observe meaningful and substantial effects.

**Table 4:** Three separate regression analyses to test the hypotheses

*Dependent variables*									
	Complaint satisfaction (n = 126)			Overall satisfaction with company (n = 124)			Trust in company (n = 127)		
	**B**	***b***	***p *-value**	**B**	***b***	***p *-value**	**b**	**B**	***p *-value**

*Independent variables*									
Functional quality	0.28	0.39	< .001	0.53	0.5	< 0.001	0.52	0.52	< 0.001
Technical quality	0.69	0.58	< .001	0.06	0.04	0.468	0.13	0.21	0.011
Total variance (R^2^) explained	R^2 ^= 76% *p *< 0.001			R^2 ^= 32% *p *< 0.001			R^2 ^= 43% *p *< 0.001		

Lastly, we included age, sex, and length of the relationship between client and insurer as control variables in the regression analyses. The inclusion of these variables did not affect the results and we also did not find any significant effect of these variables on consumers' satisfaction evaluations and trust in the company.

### Further analysis

Since technical quality does not have a direct effect on overall satisfaction, we explored further if complaint satisfaction might be mediating the relationship between technical quality and overall satisfaction. We followed the approach as suggested by Baron and Kenny [38] to test for mediation. First, we regressed overall satisfaction on technical quality only. The estimates show that technical quality is now significant (*p *< 0.001). Second, we regressed complaint satisfaction with technical quality and we also found a significant effect (*p *< 0.001). Lastly, we regressed overall satisfaction on technical quality and complaint satisfaction and found that there is no effect of technical quality on overall satisfaction when controlling for complaint satisfaction. Thus, we can conclude that complaint satisfaction acts as a mediator between technical quality and overall satisfaction. We followed the same approach to explore if complaint satisfaction might be mediating the relationship between functional quality and overall satisfaction. We do not find support for this mediation. This illustrates that an increase in overall satisfaction can be achieved by improving functional quality directly.

## Discussion

Overall, the proposed conceptual model to investigate consumers' evaluation of complaint handling in the health insurance market is supported by our data.

### Main effects

More specifically, we find support for all but one of the main effects as put forward in hypotheses H1 and H2. Namely, technical quality has no effect on overall consumer satisfaction. We did anticipate that functional quality would be a better predictor of overall satisfaction as compared to technical quality based on the argument that consumers evaluate elements of functional quality not only for a specific transaction, but for various transactions over a longer period. Yet, the fact that technical quality does not have any effect on overall satisfaction might be specific to the context of complaint management, a situation in which a consumer contacts a company to resolve a specific issue. Therefore, consumers might be particularly focused on the specific outcome they received and not on judging the company as a whole. Moreover, we found that functional and technical quality have a positive impact on trust in the company (H2). Thus, by focusing and improving elements of functional and technical quality, managers can increase consumers' trust in the company. This is important, since research has shown that consumers who trust a company show it more commitment and in turn intend to remain a customer [34]. Thus, by improving elements of technical and functional quality, managers can increase consumers' trust in their company and in turn focus on building long-term relationships with customers, which is crucial in a competitive market. The results illustrate that elements of the functional and technical quality of service delivery during the handling of complaints are crucial if managers are to improve complaint satisfaction, as well as trust in the company. Yet, overall satisfaction with the company seems only to be affected by functional elements.

### Relative effects

In addition to direct effects, we hypothesized that there is a difference between functional and technical quality in the degree of the effect on satisfaction measures (H3). This is suggested by our data. Functional quality has a stronger effect on overall satisfaction than technical quality and technical quality has a stronger effect on complaint satisfaction than functional quality. These findings are theoretically and managerially relevant since they provide evidence that there is a difference between complaint satisfaction and overall satisfaction. Therefore, it is crucial to know what a company is interested in. Does the company focus on improving complaint satisfaction or overall satisfaction with the company, or both? This in turn has consequences for the measurement of satisfaction. Therefore, one might conclude that there is no effect of technical quality on consumer satisfaction *in general*. However, it is only long-term measures of satisfaction, that is cumulative transaction with a company over a period of time, that do not seem to be affected by the technical quality of the service delivered, in other words the handling of the complaint. Yet, technical quality indirectly affects the overall satisfaction through complaint satisfaction, thus making it critical to focus too on the elements of technical quality when dealing with consumer complaints. Lastly, we found that functional quality has a stronger effect on trust in the company than technical quality (H4). Again, this result might be specific to the context of our study. It might be difficult for consumers to evaluate fully the outcome of their complaint, for instance as compared to a product which does not work anymore and thus consumers might focus more on functional aspects such as the friendliness of the personnel. These results are relevant to managers for improving the aspects of functional as well as technical quality; a company is able to improve its complaint satisfaction, overall satisfaction and trust in the company.

Regarding functional elements, both, how a complaint is being handled, that is the time it takes to process, and how complainants are treated, for example staff friendliness, are aspects for managers to investigate and perhaps adjust in their procedure for handling a complaint. With respect to technical quality, it might be harder for companies to do something directly related to the outcome of a specific complaint. For example if a customer complains that the costs of treatment are not reimbursed and the company concludes that the customer is not insured, and therefore not reimbursed for this specific treatment, then there is nothing that can be done regarding the technical quality of the complaint. Yet, managers can evaluate the cause of customers' complains in such cases. There could for instance be a communication problem. For instance, customers might not be properly informed. As a result, investigating the cause of complaints is crucial.

We are also aware of the limitations of this study and recognize avenues for future research. First, we asked for respondents' evaluations retrospectively, that is based on a complaint made up to 12 months ago. We are aware that respondents' recall may be biased. Future research could restrict research to within six months [36] of the complaint or even have companies try to get in contact with consumers immediately after a complaint has been handled. Furthermore, we focus on two dimensions of service quality, while there are additional variables that might affect consumer satisfaction. For instance, how serious the complaint is might play an important role and might affect the relationship between dimensions of service quality and measures of satisfaction. Moreover, in addition to consumer satisfaction and trust measures, other relevant outcome variables of service quality should be addressed in future studies. For instance, word-of-mouth, loyalty, and commitment might be important variables worth investigating. This study focused on consumers' evaluation of complaint handling without focusing on companies' guidelines for dealing with complaining customers. Future research could investigate the specific procedures for handling complaints. For example, it could investigate specific guidelines either for dealing with the outcomes of complaints or how employees deal with customers. We expect that these procedures have a direct impact on consumers' service quality evaluations and eventually their complaint satisfaction evaluations.

## Conclusions

Overall, we found support for our model of consumers' evaluation of complaint handling in the health insurance market. Specifically, we illustrate that managers who focus on functional as well as technical quality are able to improve the company's complaint satisfaction, overall satisfaction and trust in the company. In this study we also show that the size of the effect of functional and technical quality on complaint satisfaction, overall satisfaction with the company, and trust in the company differ. Therefore, it is crucial for managers to be precise about the type of complaint satisfaction evaluation they are interested in.

## Competing interests

SW and JJ declare that they have no competing interests. EC is part-time (40%) employed by UVIT. This research is funded by the Dutch health care insurer UVIT.

## Authors' contributions

SW analyzed the data and drafted the manuscript. JJ provided expertise in the design of the study, interpreting the results and assisted in drafting the manuscript. EC contributed to the design of the study, data collection as well as revision of the manuscript. All authors have read and approved the final manuscript.

## Pre-publication history

The pre-publication history for this paper can be accessed here:

http://www.biomedcentral.com/1472-6963/11/310/prepub
